# RNA-based therapy in the management of lipid disorders: a review

**DOI:** 10.1186/s12944-022-01649-3

**Published:** 2022-04-23

**Authors:** Dirk Jacobus Blom, Adrian David Marais, Rajen Moodley, Nico van der Merwe, Alet van Tonder, Frederick Johan Raal

**Affiliations:** 1 Department of Medicine, Division of Lipidology and Hatter Institute for Cardiovascular Research in Africa, University of Cape Town, Cape Town, South Africa; 2 Division of Chemical Pathology, Faculty of Health Sciences, University of Cape Town, Cape Town, South Africa; 3 Netcare Umhlanga Medical Center, Umhlanga, KwaZulu Natal, South Africa; 4 Netcare Greenacres Hospital, Port Elizabeth, South Africa; 5 Medical Affairs, Novartis, Waterfall City, South Africa; 6 Carbohydrate and Lipid Metabolism Research Unit, Faculty of Health Sciences, University of Witwatersrand, Johannesburg, South Africa

**Keywords:** RNA-based therapies, Lipid disorders, Small interfering RNA, Antisense oligonucleotides, Lipid-lowering, LDL-C

## Abstract

This review focuses on antisense oligonucleotides and small interfering ribonucleic acid therapies approved or under development for the management of lipid disorders. Recent advances in RNA-based therapeutics allow tissue-specific targeting improving safety. Multiple potential target proteins have been identified and RNA-based therapeutics have the potential to significantly improve outcomes for patients with or at risk for atherosclerotic cardiovascular disease. The advantages of RNA-based lipid modifying therapies include the ability to reduce the concentration of almost any target protein highly selectively, allowing for more precise control of metabolic pathways than can often be achieved with small molecule-based drugs. RNA-based lipid modifying therapies also make it possible to reduce the expression of target proteins for which there are no small molecule inhibitors. RNA-based therapies can also reduce pill burden as their administration schedule typically varies from weekly to twice yearly injections. The safety profile of most current RNA-based lipid therapies is acceptable but adverse events associated with various therapies targeting lipid pathways have included injection site reactions, inflammatory reactions, hepatic steatosis and thrombocytopenia. While the body of evidence for these therapies is expanding, clinical experience with these therapies is currently limited in duration and the results of long-term studies are eagerly awaited.

## Introduction

Cardiovascular disease remains the leading cause of mortality globally, accounting for approximately 17.9 million deaths in 2016 [1]. Approximately 85% of these deaths were due to myocardial infarction or stroke. The European Society of Cardiology estimates that more than one in three of all potential years of life lost can be attributed to death from cardiovascular disease [2]. Unfavorable concentrations of plasma lipoproteins such as low-density lipoprotein (LDL), remnant lipoproteins or lipoprotein (a) (Lp(a)) contribute causally to the pathogenesis of atherosclerotic cardiovascular disease (ASCVD). While a healthy lifestyle can reduce the risk of cardiovascular disease, some individuals require medication, such as statins, to lower atherogenic lipoproteins sufficiently.

**Fig. 1:**
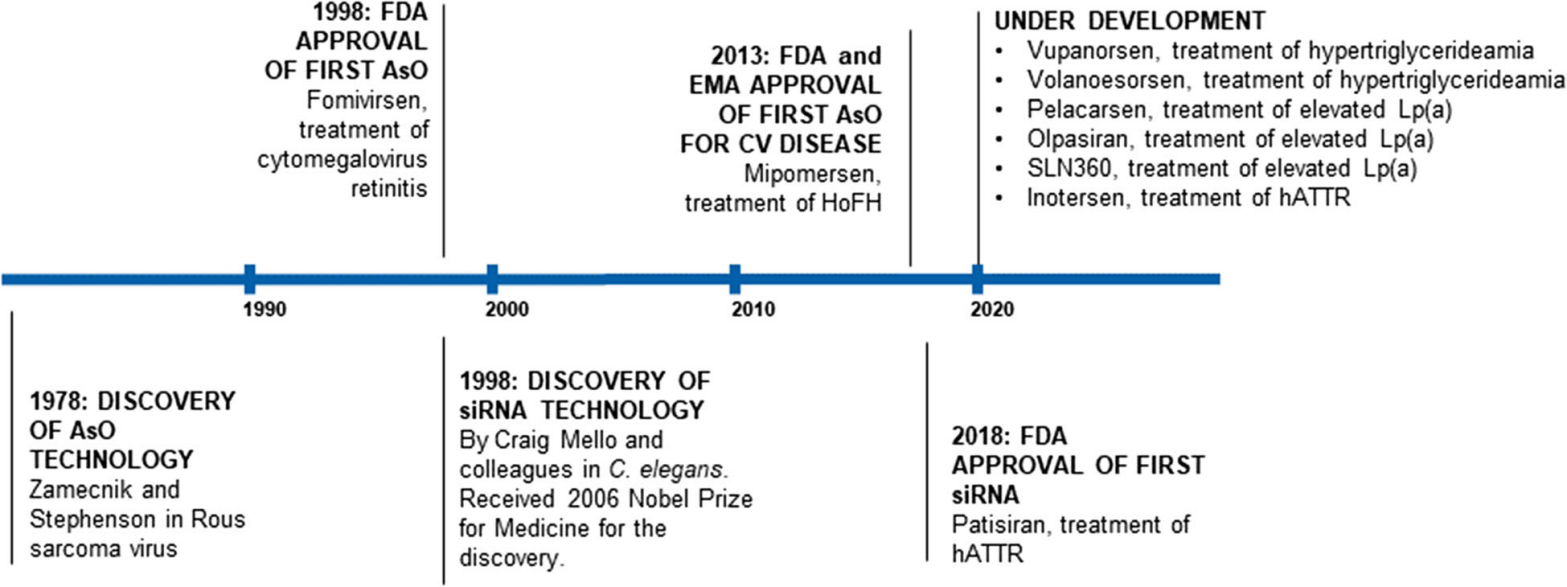
Development of RNA-based therapies for the treatment of cardiovascular and other disorders. AsO: antisense oligonucleotides; FDA: Food and Drug Administration; EMA: European Medicines Agency; CV: cardiovascular; HoFH: homozygous familial hypercholesterolemia; siRNA: small interfering ribonucleic acids; hATTR: hereditary transthyretin amyloidosis; Lp(a): lipoprotein(a)

Pharmacotherapy for cardiovascular diseases was for many years almost entirely reliant on small molecules with antibody-based therapies introduced less than 10 years ago. While these therapies are highly effective in many conditions, the identification of novel drug targets brought the need for highly targeted RNA-based therapies treatments to the fore [3]. The stability of RNA-based therapies, and concerns regarding the potential for late complications of drugs with long half-lives as well as the need to demonstrate improved clinical outcomes have resulted in an extended development period for these treatments (Fig. 1) [3].

**Fig. 2:**
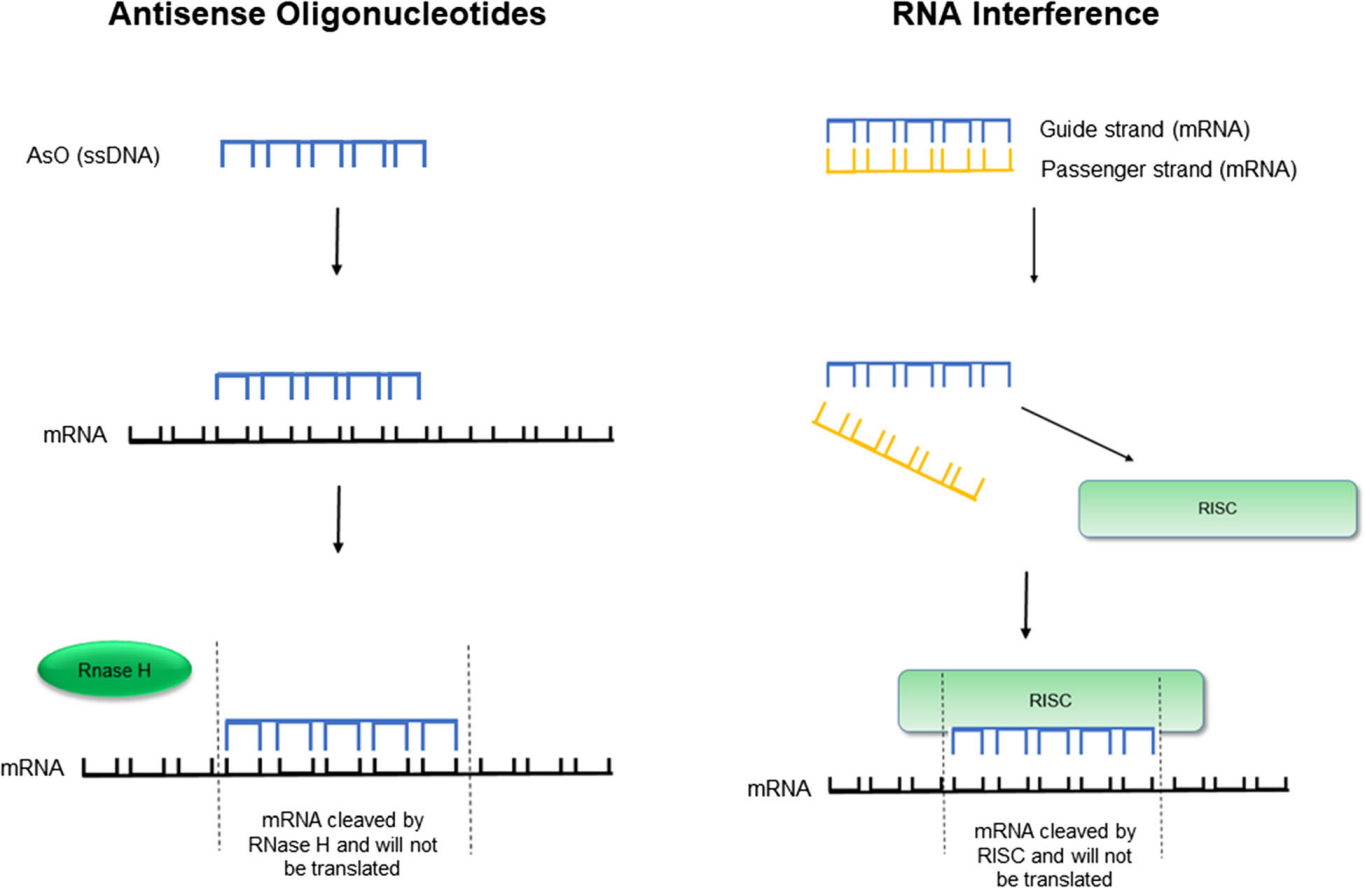
Graphic depiction of antisense oligonucleotide and RNA interference mechanism of actions

Nucleic acid-based therapies, including antisense oligonucleotides (AsO) and small interfering ribonucleic acids (siRNA) strategies, allow for selective gene silencing, therefore preventing production of proteins that may cause or exacerbate disease states. Figure 2 illustrates the mechanisms of action of AsO and siRNA therapies.

The RNase-mediated mechanism of action of AsOs is demonstrated here. AsOs are administered as chemically stabilized single stranded oligonucleotides that bind to target mRNA and thereafter recruit RNase H to cleave the target mRNA. RNA interference treatments are administered as double stranded mRNA. After administration, the passenger strand dissociates while the guide strand binds to the RNA-induced silencing (RISC) complex resulting in cleavage of target mRNA.

AsO: antisense oligonucleotides; ssDNA: single strand deoxyribonucleic acid; mRNA: messenger ribonucleic acid; RISC: RNA-induced silencing complex.

While both these strategies silence the expression of a particular gene, there are important differences between them. AsOs are chemically stabilized, single-stranded oligonucleotides that that selectively inhibit the translation of mRNA through a variety of mechanisms, including RNase H1-mediated cleavage of mRNA as illustrated above, alteration of pre-mRNA splicing and modulation of mRNA translation [4–7]. siRNA therapeutics, on the other hand, are double stranded RNA fragments that are incorporated into the RNA-induced silencing complex (RISC) resulting in selective cleaving of mRNA [6, 7]. The resulting cleaved mRNA sequences are not translated to the target protein.

FDA approval has been granted to several RNA-based therapies (Table 1). Several other RNA-based therapies are currently undergoing clinical development and show potential as treatment for cardiovascular diseases.

**Table 1:** FDA-approved RNA-based therapies

Molecule	Approval date	Therapeutic area
Fomivirsen [8]	1998	Cytomegalovirus retinitis
Pegaptinib [9]	2004	Neovascular age-related macular degeneration
Mipomersen [9]	2013	Familial hypercholesterolemia
Eteplirsen [9]	2016	Duchenne muscular dystrophy
Defibrotide [9]	2016	Hepatic veno-occlusive disease
Patisiran [10]	2018	Polyneuropathy in hereditary transthyretin-mediated amyloidosis
Givosiran [11]	2019	Acute hepatic porphyria
Inclisiran [12]	2021	Heterozygous familial hypercholesterolemia or clinical atherosclerotic cardiovascular disease

This review focuses on AsO and siRNA therapies under development for the management of lipid disorders (Table 2).

**Table 2:** RNA-based therapies discussed in this review

RNA-based therapy	Target	Type	Modification
CiVi007 [13]	PCSK9	AsO	LNA
Inclisiran [14]	PCSK9	siRNA	GalNAc conjugate
Mipomersen [15]	Apolipoprotein B100	AsO	Ribose modified with addition of 2′-*O*-methoxyethyl
Pelacarsen [16]	Apo(a)	AsO	GalNAc conjugate
Olpasiran [17]	Apo(a)	siRNA	GalNAc conjugate
Vupanorsen [18]	ANGPTL3	AsO	GalNAc conjugate
ARO-ANG3 [19]	ANGPTL3	siRNA	GalNAc conjugate
Volanesorsen [20]	ApoC3	AsO	Ribose modified with addition of 2′-*O*-methoxyethyl
ARO-APOC3 [19]	ApoC3	siRNA	GalNAc conjugate

*LNA* locked nucleic acid, *GalNAc* N-acetylgalactosamine, *PCSK*9 proprotein convertase subtilisin/kexin type 9

### Targeting LDL

LDL is the most abundant of the ApoB-containing lipoproteins and is widely recognized as a modifiable risk factor for ASCVD [21]. Data from epidemiological studies, Mendelian randomization studies, animal studies and clinical trials show conclusively that elevated levels of LDL cholesterol (LDL-C) and duration of exposure to these elevated levels are causally related to the risk of developing atherosclerotic cardiovascular disease [22, 23]. Indeed, in a meta-regression analysis of 49 clinical trials with 312,175 participants, each 1-mmol/L (38.7-mg/dL) reduction in LDL-C level was associated with a relative risk (RR) of major vascular events of 0.77 (95% CI, 0.71 to 0.84; *P* < .001) for statins and 0.75 (95% CI, 0.66 to 0.86; *P* = .002) for established non-statin interventions that act primarily via upregulation of LDL receptor expression [24].

Current European lipid treatment guidelines suggest an LDL-C target of 1.8 mmol/L and 1.4 mmol/L for high-risk patients and very high-risk patients, respectively [25]. Additionally, LDL-C should be reduced by at least 50% from the untreated baseline [25]. While lipid-lowering therapy such as statins and ezetimibe are sufficient for goal attainment in many patients, markedly elevated LDL-C at baseline, as seen for instance in patients with familial hypercholesterolemia (FH), often prevents patients from reaching their LDL-C goal. Furthermore, poor adherence to treatment, tolerability concerns and elevated expression of PCSK9 as result of statin treatment may detract from effective management of hyperlipidemia [26]. For these patients inhibitors/neutralizers of PCSK9, such as the monoclonal antibodies alirocumab and evolocumab, or RNA-based therapy such as inclisiran may be considered [25].

### PCSK-9 targeted RNA-based therapy

#### CiVi007

##### Background information

CiVi007 is a third generation AsO targeted against *PCSK9* mRNA [13]. Administration of an AsO such as CiVi007 results in reduced hepatic production of PCSK9 protein, decreased degradation of LDL receptors (LDLR) and an increased number of LDLR on the hepatic cell surface available to bind circulating LDL [14]. The subsequent reduction in plasma LDL-C concentration is longer lasting than that achieved with traditional small molecules such as statins.

Interestingly, this molecule is under development in subcutaneous and oral formulations and a once monthly dosing schedule is expected [27].

##### Clinical development: efficacy

A phase 1, first in human, placebo-controlled, single blind, randomized study was recently completed in 36 patients with elevated LDL-C (NCT03427710). This study assessed the safety, tolerability as well as pharmacokinetic and pharmacodynamic characteristics of CiVi007. Subsequently a phase 2a, placebo-controlled, randomized study assessing safety, tolerability and pharmacokinetic and pharmacodynamic characteristics of CiVi007 in patients on concomitant statin therapy has also recently been completed (NCT04164888). Results of these studies are not currently available.

##### Clinical development: safety

Safety information for CiVi007 is currently unavailable.

#### Inclisiran

##### Background information

The siRNA inclisiran silences the intracellular translation of *PCSK9* mRNA by mimicking the physiological process of RNA interference. Inclisiran consists of a double-stranded siRNA conjugated to a synthetic ligand, GalNAc, which binds selectively to the asialoglycoprotein receptor (ASGPR) expressed mainly on the surface of hepatocytes [28, 29].

Once inclisiran is internalised into hepatocytes through endocytosis, the guide (antisense) strand dissociates from the passenger strand and enters the RISC. This complex selectively binds to *PCSK9* mRNA and cleaves it, thereby inhibiting the translation of its protein [30]. As the complex of inclisiran siRNA in the RISC complex is not readily degraded by cellular nucleases, its effect on PCSK9 levels is durable: it has been reported that PCSK9 levels are reduced by approximately 50% from baseline for up to 180 days after subcutaneous administration of 300 mg of inclisiran sodium [14, 31].

##### Clinical development: efficacy

The clinical development of inclisiran started in 2011 with a small dose finding study in healthy volunteers [32] and is currently still ongoing in the ORION and VICTORION programs [33]. At the time of publication results of the trials in the VICTORION program are unavailable. A diverse population of hypercholesterolemic patients including patients with homozygous familial hypercholesterolemia (HoFH) and heterozygous familial hypercholesterolemia (HeFH) participated in the ORION program and the results of the phase 3 studies will be briefly discussed (also see Table 3).

**Table 3:** A summary of the most recently completed clinical trials for each gene therapy discussed

Molecule	Trial name	Treatment arms	Primary outcome	Study duration	Patient population	Number of patients	Results: primary outcome	Safety reports^1^
Inclisiran (siRNA)^1^	ORION-9 [45]	Inclisiran 300 mg or placebo every six months	1. Between-group %∆ in LDL-C from baseline to day 510 2. Time-adjusted %∆ in baseline LDL-C between day 90 and day 540	18 months	HeFH	482	1. -47.9% between-group difference in LDL-C at day 510 (95% CI, −53.5 to −42.3; *P* < 0.001) 2. -44.3% between-group difference in LDL-C between day 90 and day 540 (95% CI, −48.5 to −40.1; *P* < 0.001)	Injection site reactions, gastroenteritis, back pain and nasopharyngitis
	ORION-10 [46]	Inclisiran 300 mg or placebo every six months	1. Between-group %∆ in LDL-C from baseline to day 510 2. Time-adjusted %∆ in baseline LDL-C between day 90 and day 540	18 months	Established ASCVD	1561	1. −52.3% between-group difference in LDL-C at day 510 (95% CI, −55.7 to −48.8; *P* < 0.001) 2. -53.8% between-group difference in LDL-C between day 90 and day 540 (95% CI, −56.2 to −51.3; *P* < 0.001)	Death from cardiovascular causes, fatal or nonfatal stroke, fatal or nonfatal MI, injection site reactions, diabetes mellitus, bronchitis, dyspnea, upper respiratory tract infections
	ORION-11 [46]	Inclisiran 300 mg or placebo every six months	1. Between-group %∆ in LDL-C from baseline to day 510 2. Time-adjusted %∆ in baseline LDL-C between day 90 and day 540	18 months	Established ASCVD and ASCVD risk equivalents	1617	1. -49.9% between-group difference in LDL-C at day 510 (95% CI, −53.1 to −46.6; *P* < 0.001) 2. -49.2% between-group difference in LDL-C between day 90 and day 540 (95% CI, −51.6 to − 46.8; *P* < 0.001)	Injection site reaction, arthralgia
Mipomersen (AsO)	Phase 3 (NCT00607373) [34]	200 mg SC QW or placebo	%∆ in LDL-C levels from baseline to week 26	26 weeks	HoFH	51	−24.7% mean LDL-C change in mipomersen group 1. -3.3% mean LDL-C change in placebo group	Injection site reaction
	Phase 3 (NCT00794664) [35]	200 mg SC QW or placebo	%∆ in LDL-C from baseline to 2 weeks after last dose	26 weeks	Severe hypercholesterolemia	58	−35.9% mean LDL-C change in mipomersen group 1. + 12.5% mean LDL-C change in placebo group	Injection site reaction Flu-like symptoms
Pelacarsen (AsO)	Phase 2 (NCT03070782) [16]	20 mg QW or Q2W or Q4W, 40 mg Q4W, 60 mg Q4W or placebo	%∆ in fasting Lp(a) level from baseline to month 6	6–12 months	Established CVD and Lp(a) > 60 mg/dL	286	−72% in 60 mg Q4W group − 80% in 20 mg QW group	Injection site reaction
Olpasiran (siRNA)	Phase 1 [17]	3, 9, 30, 75, or 225 mg olpasiran once off or placebo	1. Treatment-emergent adverse events 2. Safety laboratory analytes 3. Vital signs 4. ECGs	⁓ 7 months	Lp(a) ≥ 70 nmol/L	64	−80% mean reduction in Lp(a) at Day 113	Headache Upper respiratory tract infection
Vupanorsen (AsO)	Phase 2 (NCT03371355) [36]	20 mg QW, 40 or 80 mg Q4W	%∆ in fasting triglycerides from baseline to week 24	24 weeks	Hypertriglyceridemia, Type 2 Diabetes Mellitus (T2DM), and Nonalcoholic Fatty Liver Disease	105	− 53% reduction in triglycerides in 80 mg Q4W group	Injection site reaction
ARO-ANG3 (siRNA)	Phase 1 (NCT03747224) [37, 38]	100, 200 or 300 mg ARO-ANG3 or placebo Q4W	• Number of participants with adverse events potentially related to treatment	113 days	Healthy volunteers, FH and severe hypertriglyceridemia	94	−90% reduction in ANGPTL3 in healthy volunteers and patients with FH − 43% reduction in TG in FH patients	Headache, injection site reaction, upper respiratory tract infection
Volanesorsen (AsO)	APPROACH [39]	Volanesorsen 300 mg QW or placebo	• %∆ in fasting triglyceride level from baseline to 3 months	52 weeks	Familial chylomicronemia syndrome	66	−77% or − 19.3 mmol/L in fasting TG from baseline to month 3 (95% CI, 15.0–23.6 mmol/L, *P* < 0.001)	Injection site reaction, thrombocytopenia
	COMPASS [40]	Volanesorsen 300 mg QW or placebo	• %∆ in fasting triglyceride level from baseline to 3 months	26 weeks	Hypertriglyceridemia	113	− 72.7 ± 17.4% in TG from baseline to month 3	Injection site reaction
	BROADEN [41]	Volanesorsen 300 mg QW or placebo	• ∆ in fasting triglycerides from baseline	52 weeks	Familial partial lipodystrophies	40	−88% in fasting TG in volanesorsen group −22% in fasting TG in placebo group	Injection site reaction, thrombocytopenia
Olezarsen (AsO)	Phase 2 (NCT03385239) [42]	10 or 50 mg Q4W, 15 mg Q2W, 10 mg QW	• %∆ in fasting triglyceride level from baseline to 6 months	12 months	Established ASCVD and hypertriglyceridemia	114	−60% in fasting TG with 10 mg QW and 50 mg Q4W vs 6% increase in placebo group	Injection site reaction
ARO-APOC3 (siRNA)	Phase 1 (NCT03783377) [40, 41, 43]	10, 25 or 50 mg ARO-APOC3 or placebo	• Number of participants with adverse events potentially related to treatment	113 days	Healthy volunteers, hypertriglyceridemia and familial chylomicronemia syndrome	80	− 72% in TG for healthy volunteers −78% in TG for hypertriglyceridemia and familial chylomicronemia	Injection site reaction, headache, upper respiratory tract infection and two cases of transient elevated ALT

Data from the most advanced trials in the clinical development program were included here

The efficacy of inclisiran in patients diagnosed with HoFH was investigated in the ORION-2 and the ongoing ORION-5 studies [44]. The proof-of-concept ORION-2 single-arm, open-label, multicenter study enrolled four HoFH patients who received inclisiran sodium 300 mg as add-on to background lipid-lowering therapy consisting of high-intensity statins and ezetimibe. PCSK9 was reduced in all patients (− 40.2 to − 80.5% at day 180), while LDL-C reductions of − 17.5 to − 37.0% were reported for patients B, C and D at day 180, and no effect was observed in patient A. Patient A had a history of poor responses to both alirocumab and evolocumab [44]. As inclisiran and monoclonal antibodies directed against PCSK9 both work by indirectly upregulating LDL receptors, inclisiran is unlikely to be effective in patients with HoFH who do not have residual LDL receptor function. Based on the efficacy and safety reported in ORION-2, the ORION-5 study was initiated in 2019 and is currently ongoing (NCT03851705).

Results from three phase 3 studies, ORION-9, − 10 and − 11, were published recently [45, 46] and all demonstrate the efficacy of inclisiran in lowering LDL-C over a period of 510 days. In these studies, patients were randomized to receive inclisiran (300 mg SC) or placebo on days 1, 90, 270 and 450 as add-on to statin and/or ezetimibe treatment. A between-group difference in LDL-C of − 47.9% (95% CI, − 53.5 to − 42.3; *P* < 0.001) was observed at day 510 in patients with HeFH in ORION-9 [28]. Ray and colleagues (2020) [46] reported a between-group difference in LDL-C of − 52.3% (95% CI, − 55.7 to − 48.8; P < 0.001) at day 510 in patients with established ASCVD in the ORION-10 study. Similarly, a between-group difference of − 49.9% in LDL-C at day 510 (95% CI, − 53.1 to − 46.6; P < 0.001) was reported in a patient population with established ASCVD or ASCVD risk equivalents in ORION-11 [45]. The ORION-8 study, an open-label extension of ORION-9, − 10 and − 11, evaluating long-term safety is currently ongoing (NCT03814187).

Two cardiovascular outcomes trials (CVOT), ORION-4 (NCT03705234) and VICTORION-2 PREVENT (NCT05030428), comparing inclisiran to placebo in patients with established ASCVD with background lipid-lowering therapy are currently ongoing.

##### Clinical development: safety

Overall, inclisiran is well tolerated [32, 45, 46]. Adverse events reported by patients receiving inclisiran included injection site reactions, headache [32], nasopharyngitis, back pain, bronchitis and upper respiratory tract infections [45, 46]. Of these, mild and moderate injection site reactions are the most noteworthy. Antidrug antibodies were detected in fewer than 2.6% of patients but did not appear to affect pharmacokinetic variables or efficacy [45, 46].

In the ORION-10 study, a risk ratio of 0.7 (95% CI, 0.5 to 1.0) was reported for the prespecified exploratory cardiovascular endpoint, which included death due to CV causes, cardiac arrest, nonfatal myocardial infarction and stroke [46]. The study investigators highlighted that the overall number of events was too limited to make firm conclusions regarding improvements in cardiovascular endpoints [46].

#### Additional LDL-C-lowering therapeutic options under development

Vupanorsen, a second generation AsO [18], and ARO-ANG3, an siRNA [37], both selectively inhibit hepatic translation of ANGPTL3 mRNA and are currently under development for the management of elevated triglycerides and LDL-C. These molecules are described in greater detail in the section on drugs interfering with the metabolism of triglyceride-rich lipoproteins.

### Apo-B targeted RNA-based therapy

The apolipoprotein B (*ApoB*) gene is located on chromosome 2 and contains 29 exons [18]. ApoB RNA editing results in two distinct forms of apoB: ApoB100 and ApoB48 [47]. ApoB100 is expressed in the liver and is the structural apoprotein of very-low-density lipoproteins (VLDL) and ultimately LDL, while ApoB48 is expressed in the intestinal tract and is essential for the formation of chylomicrons (CM) [48].

ApoB100 is found in VLDL, IDL, LDL and Lp(a). Each lipoprotein particle contains a single apoB100 molecule. ApoB100 binds to the LDLR allowing for the clearance of ApoB-containing lipoproteins from the circulation. Inhibition of ApoB production (either in the liver or intestines) would result in reduced export of either VLDL and/or chylomicrons providing a lipid-lowering mechanism that is independent of receptor mediated lipoprotein clearance.

#### Mipomersen (ISIS301012)

##### Background information

The AsO, mipomersen, selectively silences the mRNA responsible for the coding of ApoB100, thereby reducing ApoB100 concentrations [34].

Mipomersen is administered at a dose of 200 mg once weekly and has shown LDL-C reductions of approximately 30% in various patient populations [34, 35, 49].

##### Clinical development: efficacy

Raal and colleagues (2010) [34] conducted a multicenter, randomized, double-blind study to investigate the efficacy of mipomersen at 200 mg once weekly in 49 patients with HoFH over a 26-week period. Mean reductions in LDL-C from baseline were 24.7% for mipomersen, and 3.3% for placebo (*P* = 0.0003) [34]. The response to mipomersen was highly variable, ranging from a decrease of 82% to an increase of 2%.

The efficacy of mipomersen in patients with severe hypercholesterolemia (LDL-C at baseline ≥5.1 mmol/L) was evaluated in a randomized, multicenter, double-blind study comparing mipomersen 200 mg SC weekly with placebo in 58 patients on background lipid-lowering therapy [35]. At the end of the 26-week study period, LDL-C levels were reduced by 35.9% with mipomersen treatment (baseline LDL-C of 7.2 mmol/dL) and by 12.5% with placebo treatment (baseline LDL-C of 6.5 mmol/dL) (*P* < 0.001) [35]. Stein et al., (2012) [50] conducted a similar 26-week, multicenter, double-blind, randomized study in patients with HeFH and coronary artery disease on background statin therapy. An LDL-C reduction of 28% was demonstrated with mipomersen treatment, compared with 5.2% for placebo (P < 0.001) [50]. A systematic review and meta-analysis of mipomersen trials is presented in [51].

Key mipomersen studies are briefly summarized in Table 3.

##### Clinical development: safety

The most commonly reported adverse events during the clinical development program of mipomersen included injection site reactions (erythema, pruritus, pain and post-inflammatory hyperpigmentation), fatigue, pyrexia, chills, malaise, myalgia, and arthralgia [34, 35, 49]. Hepatic AEs (alanine aminotransferase (ALT) elevations and hepatic steatosis) were common in the mipomersen arm. The incidence of renal adverse events was similar across both the mipomersen and placebo arms [35]. The occurrence of cardiac events, including angina, MI, cardiac failure, CAD, and supraventricular extrasystoles was higher in the mipomersen arm (12 events vs one event) [35].

Based on these results the FDA issued a black box warning for mipomersen, stipulating increased risk for hepatotoxicity due to the association of mipomersen with elevated ALT and hepatic steatosis [50]. Hepatotoxicity was noted as early as 6 months after treatment initiation [52]. The European Medicines Agency did not grant mipomersen marketing approval because of concerns regarding high dropout rates from clinical trials over a period of 2 years, hepatic toxicity and increased cardiovascular event rates [53]. Marketing of mipomersen has subsequently been discontinued.

### Lp(a)-targeted RNA-based therapy

Lp(a) consists of one LDL particle covalently bound to apolipoprotein(a) (apo(a)). Lp(a) levels are predominantly genetically determined and may vary several hundredfold amongst individuals. Loop-like structures, referred to as kringles, are present on apo(a) in variable numbers [54]. Genetic studies have demonstrated an association between the number of kringle IV type 2 repeats and the risk for coronary heart disease [55]. Lp(a) is rich in oxidized phospholipids and has proatherogenic, proinflammatory and prothrombotic effects and is recognized as an inherited, independent and causal risk factor for cardiovascular disease [56, 57].

Lp(a) values > 50 mg/dL in Caucasians are associated with increased cardiovascular risk [58]. Currently there are no approved pharmacological therapies that target Lp(a). The only highly effective option for lowering Lp(a) is lipoprotein apheresis which results in a time-averaged 30–35% reduction in Lp(a) [59]. Treatment with niacin or PCKS9 mAbs lowers Lp(a) modestly by 20–30% [60, 61] which is unlikely to provide significant clinical benefit as a Mendelian randomization analysis indicates that large absolute reductions in Lp(a) are likely required to meaningfully reduce the risk of coronary artery disease [62].

#### Pelacarsen (TQJ230/AKCEA-APO(a)-LRx/IONIS-APO(a)-LRx)

##### Background information

The AsO pelacarsen selectively inhibits the production of apo(a) by targeting *LPA* mRNA. Conjugation to the synthetic GalNAc ligand ensures selective inhibition of hepatic apo(a) synthesis, the main site of Lp(a) synthesis [16, 28, 29].

The extended half-life of pelacarsen of approximately 1 month translates into infrequent administration.

##### Clinical development: efficacy

Several clinical studies evaluating the efficacy and safety of pelacarsen have been completed. A dose-ranging phase 2 trial was conducted in patients with established CVD and Lp(a) > 60 mg/dL. Patients were randomly assigned to treatment with varying doses of pelacarsen (20 mg QW or Q2W or Q4W, 40 mg Q4W, 60 mg Q4W) over a six-month period [63]. Reduction in Lp(a) levels of 72% was observed in patients receiving 60 mg Q4W while patients treated with 20 mg QW showed an 80% reduction in Lp(a) (Table 3). This effect became apparent by Week 4, with near maximal effect reached by Week 16 after the last dose [16].

A phase 3 CVOT (Lp(a) HORIZON, NCT04023552) evaluating the effect of pelacarsen on major cardiovascular events in patients with Lp(a) ≥ 70 or ≥ 90 mg/dL is currently underway.

##### Clinical development: safety

Thus far, clinical experience with pelacarsen indicates that the treatment is well-tolerated. The most common adverse events reported to date are injection site reactions, flu-like symptoms, headache, urinary tract infections and fatigue [16]. No marked effects on liver function, renal function or platelet count have been reported [16].

#### Olpasiran (AMG890)

##### Background information

Olpasiran is an siRNA which selectively inhibits the transcription of *LPA* mRNA restricting production of Lp(a) [17, 63].

Studies indicate sustained Lp(a) reduction of up to 80% for 6 months after administration of a single dose of olpasiran [17].

##### Clinical development: efficacy

Results from a phase 1 study in 64 healthy adults demonstrated that a single dose of olpasiran at 9 or 75 mg reduced Lp(a) levels after 43 days by 75 and 89%, respectively [64] (refer to Table 3). Phase 1 and 2 clinical trials are presently ongoing to determine the safety and tolerability of olpasiran in patients with elevated plasma Lp(a) (NCT03626662, NCT04270760). Primary completion for the phase 2 study (NCT04270760) was reached in December 2021; however, at time of submission full results were not yet available.

##### Clinical development: safety

Adverse events reported during the phase 1 study included headache (10% AMG890, 25% placebo) and upper respiratory tract infections (15% AMG890, 13% placebo). No serious safety concerns were identified [17].

### Triglyceride-rich lipoprotein targeted RNA based therapy

It was previously thought that reduced levels of high-density lipoprotein (HDL-C) are causally associated with increased cardiovascular risk. However, an inverse relationship between HDL-C and triglycerides (TG) exists [65, 66] and new evidence suggest that hypertriglyceridemia and the concomitant increase in remnant cholesterol, rather than low levels of HDL-C, are causally linked to cardiovascular disease [67, 68].

Remnant cholesterol is defined as the cholesterol content of all triglyceride-rich lipoproteins, i.e., chylomicron remnants, VLDL, and intermediate-density lipoproteins (IDL) in the fasting or non-fasting states [68]. Triglyceride-rich lipoproteins are larger than LDL and carry 5–20 times more cholesterol per particle [69]. Triglycerides in triglyceride-rich lipoproteins are enzymatically degraded by various lipases such as lipoprotein lipase and hepatic lipase resulting in the formation of smaller, denser lipoproteins enriched in cholesterol. Cholesterol in these remnant lipoproteins readily accumulates in intimal foam cells contributing to plaque formation [68]. Dysregulation of triglyceride metabolism is associated with increased cardiovascular risk and therefore presents a potential therapeutic target. Indeed, results from the LURIC study demonstrated that low levels of hepatic lipase, the enzyme responsible for facilitating clearance of triglycerides from intermediate-density lipoprotein (IDL) to form LDL, are associated with increased cardiovascular risk [70].

Mendelian randomization studies have demonstrated that modulation of lipoprotein lipase (LPL) levels reduces cardiovascular risk [71]. In addition, other genes affecting triglyceride metabolism and thus cardiovascular risk have also been identified: *APOA5, APOC3* and *TRIB1* [67]. Two of these genes are currently being investigated as potential targets for modulating triglyceride metabolism using nucleic acid-based therapies: angiopoietin-like protein 3 (ANGPTL3) and apolipoprotein C3 (apoC3).

Angiopoietin-like protein 3 (ANGPTL3) is expressed and secreted by the liver and affects lipolysis and clearance of triglyceride-rich lipoproteins and remnants through reversible inhibition of LPL, inhibition of endothelial lipase and activation of lipolysis in adipocytes [18, 72]. Genetic studies have demonstrated that individuals with loss-of-function mutations in *ANGPTL3* have extremely low levels of most plasma lipoproteins and a reduced risk of coronary artery disease [73, 74]. ANGPTL3 levels in the circulation can be reduced with monoclonal antibodies, such as evinacumab, or by reducing production either with an antisense oligonucleotide or small interfering RNA approach. ANGPTL4 is also involved in the regulation of lipolysis and clearance of triglyceride-rich lipoproteins and is thus a potentially promising target but *ANGPTL4-*null mice display an adverse phenotype characterized by growth arrest, anorexia, intestinal fibrosis and ultimately death making ANGPTL3 the more attractive target [18, 75].

Apolipoprotein C3 (apoC3) is an apoprotein found predominantly on triglyceride-rich lipoproteins and inhibits lipoprotein lipase mediated lipolysis [76], thereby increasing plasma triglyceride levels. Additionally, apolipoprotein C3 is implicated in impairing the hepatic uptake and degradation of triglyceride-rich lipoproteins by LDL receptors [77], suggesting that inhibition of apoC3 may reduce residual cardiovascular risk. Indeed, genome-wide association studies have demonstrated that a null mutation in the *APOC3* gene results in reduced expression of apolipoprotein C3, extremely low levels of triglycerides and reduced risk of cardiovascular disease [78–80].

Treatment goals specific for triglyceride levels in cardiovascular risk reduction have not yet been set in all lipid guidelines, though the American College of Cardiology recently published a consensus treatment pathway for patients with ASCVD and persistent hypertriglyceridemia [25, 81]. If TG remain persistently elevated (> 1.7 mmol/L) despite lifestyle modification pharmacological intervention should be considered. Several therapies are currently available to lower TG levels including fibrates, icosapent ethyl and statins [25] while four RNA-based therapies with potent TG reducing potential are under development.

#### Vupanorsen (IONIS ANGPTL3-L_RX_/AKCEA-ANGPTL3-L_RX_/ISIS 703802)

##### Background information

Vupanorsen is a second generation AsO selective for ANGPTL3 mRNA and is conjugated to GalNAc to ensure selective hepatic uptake [18]. A advantage of the AsO strategy is that immune responses, which may be induced by monoclonal antibodies, are avoided [72].

Vupanorsen has been investigated as a weekly or monthly dose and TG lowering more than 60% has been reported [36, 82].

##### Clinical development: efficacy

The results of a double blind, placebo-controlled, randomized phase 1 study have been published [82]. Forty-four healthy participants were randomly assigned to receive a single dose of ANGPTL3-L_RX_ (20, 40, or 80 mg), multiple doses (10, 20, 40, or 60 mg once weekly) or placebo over a six-week period. In patients receiving a single injection (*n* = 9 vupanorsen, *n* = 3 placebo), dose dependent decreases in ANGPTL3 protein, triglycerides, VLDL cholesterol, non-HDL cholesterol and total cholesterol were observed in the vupanorsen treatment group. In patients receiving multiple doses of vupanorsen (*n* = 24 active, *n* = 8 placebo), significant, dose dependent reductions in ANGPTL3 protein levels were observed at day 43 [82]. Patients receiving the highest dose of vupanorsen (60 mg) showed a reduction of almost 85% in ANGPTL3 protein levels as compared to baseline. A substantial 63.1% reduction in triglycerides from baseline was observed [82].

A phase 2 study investigated vupanorsen given subcutaneously at doses of 40 or 80 mg once monthly or 20 mg once weekly in 105 patients with elevated fasting plasma TG, a confirmed diagnosis of type 2 diabetes, hepatic steatosis and BMI between 27 and 40 kg/m^2^ [36]. After a 6-month treatment period, the greatest reduction in triglyceride levels (53% reduction from baseline) and ANGPTL3 levels (62% reduction from baseline) was reported in the group receiving 80 mg vupanorsen once monthly [36]. This study is summarised in Table 3.

Several small studies assessing vupanorsen’s efficacy and safety in patients with familial partial lipodystrophy (NCT03514420), familial chylomicronemia syndrome (NCT03360747) and patients diagnosed with type 2 diabetes, hypertriglyceridemia and nonalcoholic fatty liver disease (NCT03371355) have recently been completed and preliminary results indicate reduction in fasting triglycerides ranging from 33 to 60%.

Another dose-ranging study (TRANSLATE-TIMI 70; NCT04516291) as well as a phase 1 study in healthy, adult Japanese participants (NCT04459767) have been completed, however results are yet to be published. A study evaluating the safety and efficacy of vupanorsen in patients with familial hypercholesterolemia (NCT02709850) is ongoing.

##### Clinical development: safety

No serious adverse events were reported during the phase 1 study [82]. Adverse events recorded in the multiple-dose arm and included headache (three reports) and dizziness (three reports) [82]. Adverse events reported during the phase 2 study were mostly mild in nature and included injection site reactions (20.5% of all vupanorsen-treated patients), urine protein/creatinine ratio > 325 mg/g (11.5% of vupanorsen-treated patients) and urine albumin/creatinine ratio > 165 mg/g (9.0% of all vupanorsen-treated patients) [36].

Pfizer announced in January 2022 that they would be returning the rights for vupanorsen to IONIS following a careful review of the TRANSLATE-TIMI 70 results. Pfizer stated that ‘the study met its primary endpoint, achieving a statistically significant reduction in non-high density lipoprotein cholesterol (non-HDL-C) — as well as statistically significant reductions in triglycerides (TG) and angiopoietin-like 3 (ANGPTL3). However, the magnitude of non-HDL-C and TG reduction observed did not support continuation of the clinical development program for CV risk reduction or severe hypertriglyceridemia’. Vupanorsen was also associated with dose-dependent increases in liver fat, and higher doses were associated with elevations in alanine aminotransferase (ALT) and aspartate aminotransferase (AST) [83].

#### ARO-ANG3

##### Background information

The siRNA therapy, ARO-ANG3, selectively inhibits hepatic translation of ANGPTL3 mRNA. ARO-ANG3 is conjugated to GalNAc ensuring selective binding to the hepatic ASGPR only [19].

Available data suggest potent reduction in systemic ANGPTL3 after monthly administration of ARO-ANG3 [37].

##### Clinical development: efficacy

Studies in non-human primates demonstrated that a single dose of 2 mg/kg resulted in a maximum reduction in systemic ANGPTL3 levels of 75%. This effect was sustained over a period of 7 weeks [84].

Preliminary results for a phase 1 study in healthy adult volunteers or patients with dyslipidemia, including familial hypercholesterolemia and severe hypertriglyceridemia, indicate potent and prolonged inhibition of ANGPTL3 levels (up to 93%) after two doses of ARO-ANG3 [37, 38]. Interestingly, the mean reduction in LDL-C, TG and ANGPTL3 observed in these studies were similar in healthy volunteers and patients with heterozygous familial hypercholesterolemia. Full results of this study have not yet been published.

Further details are shown in in Table 3.

##### Clinical development: safety

Headache, upper respiratory tract infections and injection site reactions were the most commonly reported adverse events during the phase 1 study for ARO-ANG3. No serious adverse events were reported in the preliminary results [27, 38].

#### Volanesorsen and olezarsen

##### Background information

Volanesorsen is a second generation AsO that selectively inhibits apoC3 synthesis [20]. A second- generation, GalNAc-conjugated version of volanesorsen, olezarsen, is also currently under development [85].

Studies reported reduction in TG levels of up to 53% in response to once weekly or once monthly administration of volanesorsen [36]. Reduction in TG levels up to 60% has been reported after monthly administration of olezarsen [42].

##### Clinical development: efficacy

A phase 1 double blind, placebo-controlled, dose escalation study (ISIS 304801-CS1) was conducted in 33 healthy volunteers [20]. Volunteers assigned to the multiple dose cohorts received six doses of volanesorsen at 50, 100, 200 or 400 mg over a five-week period. TG levels were reduced by up to 40% [20].

The efficacy of volanesorsen in patients with familial chylomicronemia syndrome (FCS) was investigated in the phase 3, randomized, double-blind, placebo-controlled APPROACH study [39]. A total of 66 patients were randomized to receive volanesorsen at 300 mg weekly or placebo over a 52-week period (please see Table 3). Triglyceride levels were lowered by 77% in the volanesorsen-treated group at month three, corresponding to a mean reduction of 19.3 mmol/L [39]. After 3 months of treatment, apoC3 levels were reduced by more than 80%. After completion of the APPROACH study, patients had the option of enrolling in the APPROACH open label extension (OLE) study (NCT2658175). Results for the OLE study are not yet available.

Results from the phase 3 randomized, double-blind, placebo-controlled COMPASS study indicated that volanesorsen at a dose of 300 mg once weekly reduced TG levels by a mean of 72.7 ± 17.4% from baseline to month 3 in 75 patients with predominantly non-FCS hypertriglyceridemia [40]. These effects were sustained to study end at 26 weeks.

The phase 2/3 BROADEN study (NCT02527343) evaluated the efficacy of volanesorsen in patients with partial lipodystrophy [41]. After a 52-week period, an 88% reduction in fasting triglycerides was reported for patients randomized to weekly administration of 300 mg volanesorsen with a 22% reduction in patients receiving placebo [41]. A two-year open-label extension study (NCT02639286) is planned.

Volanesorsen therapy was associated with increases in LDL-C of 135.6 and 95.5%, respectively in the APPROACH and COMPASS studies. However, increases in apoB, and thus particle numbers, were much smaller at 19.5 and 5.8%, respectively and the baseline LDL-C was low in both studies [39, 40]. Clinicians should re-evaluate lipids carefully following initiation of therapy with volanesorsen and consider whether a statin is required for LDL-C reduction or not.

A phase 2 dose-ranging study with olezarsen in patients with established ASCVD and elevated triglyceride levels was recently completed [42]. A reduction in mean triglyceride levels of up to 60% were reported for both weekly and monthly administration of olezarsen.

##### Clinical development: safety

Volanesorsen is mostly well-tolerated except for the occurrence of severe thrombocytopenia in some patients with FCS during the APPROACH study. The most commonly reported adverse event was mild injection site reaction (52% of volunteers in the Phase 1 study, 61% of patients in the APPROACH study and 23.5% of patients in the COMPASS study) [20, 39, 40]. In the APPROACH study severe thrombocytopenia (< 25,000 platelets per microliter) developed in two patients and subsequent cases of thrombocytopenia resulted in treatment discontinuation in nine patients [39]. Thrombocytopenia leading to treatment discontinuation was not reported in the COMPASS study [40].

Other adverse events reported during the clinical development program of volanesorsen included transient elevation of C-reactive protein [20], abdominal pain [39] and one potentially related case of serum sickness that occurred 2 weeks after the final dose [40].

Mild erythema at the injection site was the most frequently reported adverse event for patients treated with olezarsen [42].

#### ARO-APOC3

##### Background information

The siRNA ARO-APOC3 selectively silences apoC3 expression in the liver as it is conjugated to GalNAc which binds to the hepatic ASGPR [19].

Studies are currently investigating monthly administration of ARO-APOC3 and a marked reduction in apoC3 levels was reported [43].

##### Clinical development: efficacy

A phase 1 study to investigate the safety, tolerability, pharmacokinetics and pharmacodynamics of ARO-APOC3 is currently underway (see Table 3). Healthy volunteers as well as patients with severe hypertriglyceridemia and familial chylomicronemia syndrome were included in this dose-finding study. Preliminary results indicate that apoC3 levels were reduced between 60 and 90% for up to 10 weeks while TGs were reduced by 72% for up to 8 weeks after two doses of 50 mg of ARO-APOC3 in healthy volunteers [43]. Similar efficacy was observed in participants with hypertriglyceridemia: a mean reduction in TG of 78% was observed 4 weeks after treatment with ARO-APOC3 at a dose of 50 mg [86]. In patients with multifactorial chylomicronemia a mean reduction of 97% in TG levels were observed a month after ARO-APOC3 was administered [87]. Full results are yet to be published.

##### Clinical development: safety

No serious adverse events were reported during the phase 1 study. However, injection site reactions, headache, upper respiratory tract infection and transient alanine aminotransferase elevation in two patients with familial chylomicronemia were reported [43, 86, 87].

## Discussion

During the last century the ability of clinicians to prevent cardiovascular disease or to reduce morbidity and mortality in patients with clinically manifest disease has improved significantly leading to hopes that coronary artery disease may be eradicated by the end of the twentieth century [88]. Unfortunately, these lofty ambitions have not been realized and cardiovascular disease remains the leading cause of mortality worldwide [1].

Limitations of current lipid-modifying therapies including tolerability concerns, poor adherence, lack of options for therapeutic targets such as Lp(a) and funder-associated access restrictions are challenges that must be addressed to reduce the burden of cardiovascular disease.

RNA-based therapies have the potential to address several of these unmet needs as they can be directed highly specifically at targets and pathways that were either previously not accessible to small molecule-based therapies or where such therapies were associated with significant off-target effects. The technologies employed in these novel therapies also reduce the patient’s burden of adherence through marked reduction in administration frequency. RNA-based therapies could potentially be prescribed using a population-based approach. Although the extended duration of action and infrequent dosing of many RNA-based therapies is attractive for clinicians and patients and may improve medication adherence, it may be more difficult to deal with medication-related adverse effects given the long time it will take for many of these drugs to be cleared.

Clinical studies directly comparing antisense oligonucleotide and small interfering RNA therapies are currently unavailable and the best treatment option for each target should be selected based on the available evidence considering safety, efficacy, tolerability, cost and ease of administration. Both approaches were originally associated with safety concerns: drug-induced thrombocytopenia was observed after treatment with several AsOs while peripheral neuropathy was reported after administration of the siRNA revusiran [89]. These concerns were addressed with structural modifications to improve the safety profile through enhance therapeutic specificity [89]. In terms of administration frequency, AsOs are generally administered monthly, while the extended duration of action of siRNA therapies allows for more prolonged periods between administration.

RNA-based therapies are unlikely to displace traditional small molecules. The accessibility, relatively low cost, widespread use and efficacy of small molecules means that RNA-based therapies will likely remain complementary to small molecule-based therapies except in diseases where there are no effective small molecule-based treatments. Furthermore, the cost of RNA-based therapies will likely limit widespread use. Patisiran, the first RNAi therapy approved by the FDA, is priced at an annual cost of US $451,430 – US $677,145 per patient [90]. Such costs may require manufacturers to reduce prices in addition to developing innovative funding strategies to ensure patient access and may restrict use to high and very high-risk patients only.

Future developments in the use of the Clustered Regularly Interspaced Short Palindromic Repeats (CRISPR) system may allow for even greater improvement of therapeutic strategies [91]. CRISPR therapies affect gene expression at the level of transcription, a process that precedes the target of both siRNA and AsO therapies. Thus far the technology is being explored for possible treatment of monogenic disorders, including cardiac arrhythmias caused by mutations in the *CALM1, CALM2 or CALM3* genes. Further technological advances are required to ensure selective targeting and resolve challenges with administration [91].

### Strengths and limitations of this review

This review illustrates the role various targets play in lipoprotein metabolism and how downregulation of these proteins can influence lipid metabolism and ultimately clinical outcomes. The review is limited in that it is by no means a comprehensive account of the development of RNA-based therapies in the cardiovascular landscape. In this report for instance therapies directed against hepatic TTR synthesis for the treatment of hereditary transthyretin-mediated amyloidosis (hATTR) such as the AsO inotersen [92], the Lp(a) lowering siRNA SLN360 [93] and RNA-based therapies for the treatment of heart failure of ischemic origin, CDR132L, were not discussed. Additionally, for many of the therapeutics discussed there is limited data in the public sphere as the results of many studies are not available or only available in abstract form. RNA-based therapeutics is also a very dynamic area of research and new candidate molecules are regularly announced.

## Conclusion and future perspectives

The advent of RNA-based therapies holds great promise to significantly improve outcomes for patients living with cardiovascular conditions. Although a relatively new field of clinical research, these treatment strategies offer highly selective targeting of key genes implicated in cardiovascular diseases with limited adverse effects ushering in a new era in lipid-lowering therapies. RNA-based therapeutics offer hope for patients with genetic lipid disorders such as FCS, HoFH or very high Lp(a) for which there were previously often no or limited therapeutic options. Additionally, the infrequent dosing and high efficacy of many RNA-based therapeutics may benefit many patients with less severe lipid disorders, e.g., patients with polygenic hypercholesterolemia who nonetheless have high cardiovascular risk. Some children and adolescents with milder phenotypes of HeFH could conceivably be conveniently treated with an injection of inclisiran once every 6 months without the need for a daily pill. However, the use of these therapies may well be restricted by cost resulting in limited patient access and widespread adoption can only occur when long-term safety and benefit is confirmed in clinical outcome trials.

## Data Availability

Data sharing is not applicable to this article as no datasets were generated or analyzed during the current study.

## Abbreviations

AE: Adverse event
ALT: Alanine aminotransferase
ANGPTL3: Angiopoeitin-like protein 3
ApoB: Apolipoprotein B
ApoC3: Apolipoprotein C3
ASCVD: Atherosclerotic cardiovascular disease
ASGPR: Asialoglycoprotein receptor
AsO: Antisense oligonucleotide
BMI: Body mass index
CM: Chylomicrons
CMV: Cytomegalovirus
CRISPR: Clustered Regularly Interspaced Short Palindromic Repeats
CV: Cardiovascular
CVD: Cardiovascular disease
EMA: European Medicines Agency
FCS: Familial chylomicronemia syndrome
FDA: Food and Drug Administration
FH: Familial hypercholesterolemia
GalNAc: N-Acetylgalactosamine
hATTR: Hereditary transthyretin-mediated amyloidosis
HDL-C: High density lipoprotein
HeFH: Heterozygous familial hypercholesterolemia
HoFH: Homozygous familial hypercholesterolemia
IDL: Intermediate-density lipoprotein
LDL: Low-density lipoprotein
LDLR: Low-density lipoprotein receptor
LNA: Locked nucleic acid
Lp(a): Lipoprotein(a)
LPL: Lipoprotein lipase
mRNA: Messenger ribonucleic acid
OLE: Open label extension
PCSK9: Proprotein convertase subtilisin/kexin type 9
QW: Once weekly
Q2W: Every two weeks
Q4W: Every four weeks
RISC: RNA-induced silencing complex
RNA: Ribonucleic acid
RR: Relative risk
SC: Subcutaneous
siRNA: Small interfering ribonucleic acid
ssDNA: Single-stranded deoxyribonucleic acid
TG: Triglycerides
VLDL: Very-low-density lipoprotein
WHO: World Health Organization
